# A population-based study on health and living conditions in areas with mixed Sami and Norwegian settlements – the SAMINOR 2 questionnaire study

**DOI:** 10.3402/ijch.v73.23147

**Published:** 2014-06-18

**Authors:** Magritt Brustad, Ketil Lenert Hansen, Ann Ragnhild Broderstad, Solrunn Hansen, Marita Melhus

**Affiliations:** 1 Centre for Sami Health Research, Department of Community Medicine, UiT The Arctic University of Norway, Tromsø, Norway; 2 Department of Medicine, University Hospital of Northern Norway, Harstad, Norway

**Keywords:** Sami, epidemiology, ethnicity, health, Arctic

## Abstract

**Objectives:**

To describe the method, data collection procedure and participation in The Population-based Study on Health and Living Conditions in Areas with both Sami and Norwegian Settlements – the SAMINOR 2 questionnaire study.

**Study design:**

Cross-sectional and semi-longitudinal.

**Methods:**

In 2012, all inhabitants aged 18–69 and living in selected municipalities with both Sami and Norwegian settlements in Mid and Northern Norway were posted an invitation to participate in a questionnaire survey covering several topics related to health and living conditions. The geographical area was similar to the area where the SAMINOR 1 study was conducted in 2003/2004 with the exception of one additional municipality. Participants could alternatively use a web-based questionnaire with identical question and answer categories as the posted paper version.

**Results:**

In total, 11,600 (27%) participated (16% used the web-based questionnaire), with a higher participation rate among those over 50 (37% for women and 32% for men). Some geographical variation in participation rates was found. In addition, for those invited who also participated in the SAMINOR 1 study, we found that the participation rates increased with the level of education and income, while there was little difference in participation rates across ethnic groups.

**Conclusion:**

The knowledge generated from future theme-specific research utilizing the SAMINOR 2 database has the potential to benefit the general population in this geographical area of Norway, and the Sami people in particular, by providing knowledge-based insight into the health and living conditions of the multi-ethnic population in these parts of Norway.

Sami, Kven and Norwegian populations have traditionally inhabited Northern Norway. The SAMINOR study was designed to provide more information about this multi-ethnic population, particularly in relation to the Sami people living in Northern Norway.

The Sami are an indigenous people primarily living in the northern parts of Norway, Sweden, Finland and the Kola Peninsula in Russia. There is no direct means to estimate the number of Sami in Norway, although it is assumed that Norway has the largest proportion of the total Sami population. The first *Population-based Study on Health and Living Conditions in Areas with Both Sami and Norwegian Populations – The SAMINOR Study* was conducted in 2003/2004 (1) by the Centre for Sami Health Research, Department of Community Medicine, UiT The Arctic University of Norway in collaboration with the Norwegian Institute of Public Health, with support from the Norwegian Ministry of Health and Care Services. The data were collected in a cross-sectional epidemiological design in which all inhabitants in the selected areas aged 30 or between 36 and 79 were invited. In total, 16,968 participated, of whom approximately 35% reported a Sami affiliation. The SAMINOR 1 study consisted of questionnaire data, clinical measures and a biobank, and is described in detail elsewhere (1). The SAMINOR 1 study enabled the achievement of systematic and population-based knowledge related to selected aspects of the health and living conditions of the Sami population (2–15); such knowledge was scarce or lacking before this study was initiated.

The reasoning for a SAMINOR 2 study was mainly 2-fold: (a) the motivation to conduct a follow-up with the methodological benefits of a longitudinal design and (b) the introduction of new themes not included in SAMINOR 1.

Our aim in this paper has been to describe the method, data collection procedure and participation in the SAMINOR 2 questionnaire study.

## Methods and material

The data collection for the SAMINOR 2 study was divided into 2 steps: (a) a questionnaire-based study and (b) a clinical study including a questionnaire. This paper covers the first step, that is, the questionnaire-based data collection, referred to as the SAMINOR 2 questionnaire study.

### Sample

**Fig. 1:**
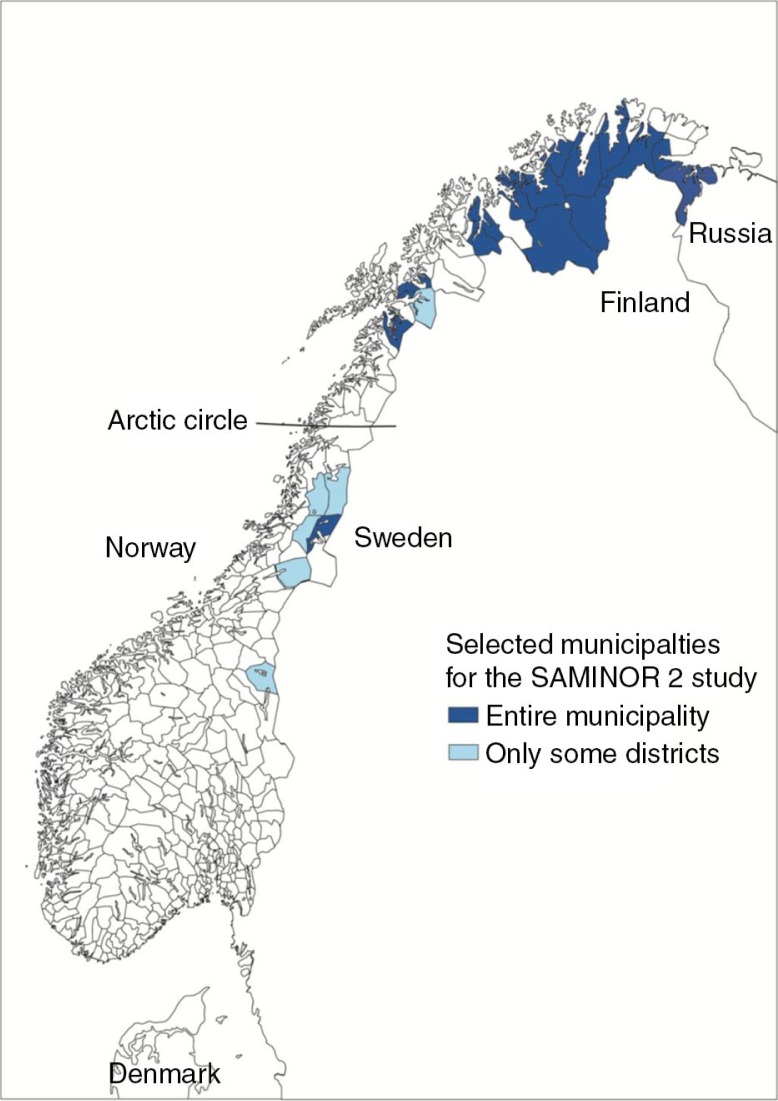
Investigation area of the SAMINOR 2 questionnaire study.

The target population for the survey was all inhabitants aged 18–69 registered in the Norwegian National Population Register by 1 December 2011 and selected from the same areas where the first SAMINOR study was carried out in 2003/2004, in addition to Sør-Varanger in eastern Finnmark. This meant that inhabitants in the following 25 municipalities were included (in some cases, only a part of the municipality was included, and indicated here in brackets): Sør-Varanger, Nesseby, Tana, Lebesby, Karasjok, Porsanger, Kvalsund, Loppa, Alta, Kautokeino, Kvænagen, Kåfjord, Storfjord, Lyngen, Lavangen, Skånland, Evenes, Narvik (Vassdalen), Tysfjord, Hattfjelldal (Hattfjelldal), Grane (Majavatn), Namskogen (Trones and Furuly), Røyrvik, Snåsa (Vinje) and Røros (Brekken) (Fig. 1). In total, 44,669 individuals were invited to participate.

### Questionnaire

The questionnaire and the information material were written in Norwegian, but also translated into 3 relevant Sami languages, Northern, Lule and Southern Sami, by professional translators. Information letters were sent out to all in Norwegian and in the Sami language relevant to the area. The Norwegian questionnaire was sent to all and a translated version in the relevant Sami language was also included for those living in the Administrative Area for the Sami Language (Nesseby, Tana, Karasjok, Porsanger, Kautokeino, Kåfjord, Lavangen, Tysfjord, Røyrvik and Snåsa).

After contributions were received from collaborating researchers from various fields, the questionnaire was coordinated and finalized by the SAMINOR study board. The questionnaire was 8 pages long and contained a set of questions corresponding to the questions used in the 2003/2004 SAMINOR data collection. This was done in order to be able to compare certain selected health and living condition variables across the period between the 2 studies. The questions regarding ethnicity were among those that were the same as the questions used in the first data collection, including family background, home language in 3 generations and self-perceived ethnicity (1).

In addition, a selection of questions was repeated from the first SAMINOR study, including some related to physical and mental health and socio-economic and living conditions. However, the majority of the questions in the questionnaire were not included in the first SAMINOR study. These new questions were included to enable assessment of new and relevant research questions, which was not possible based on the data from the former survey, but in line with the fields of interest of the researchers involved in the project.

In brief, the questionnaire had questions on self-perceived health and selected diseases, as well as health-related conditions (“The EQ-5D Health-Related Quality of Life Questionnaire”), socio-economic status, physical activity, height and weight, family and language background, mental health (“The Hopkins Symptom Checklist, or HSCL-10,” “WHO-5 Well-being Index” and “The Resilience Scale for Adults, or RSA”), tobacco and drug/alcohol use, use of hypnotics, religion, discrimination, violence and abuse, oral/dental health, suicide, gambling and health care service-related experiences, including the use of a Sami-speaking interpreter. Thus, the questionnaire covered a broad range of research topics, and a variety of formerly used questions (from comparable surveys) but also new questions.

It is beyond the scope of this paper to go in detail regarding the different questions, their validity, former use, and so on. However, we do anticipate and recommend thorough descriptions of these specific methodological issues when results from thematic research projects based on the SAMINOR data are communicated in the future.

### Logistics

The invited individuals were sampled by Statistics Norway, and each participant was assigned a unique ID code that was pre-printed on the questionnaire. Statistics Norway administrates and stores the code for future linkages, together with the participants’ personally identifiable information. The questionnaires were returned in stamped, self-addressed envelopes to the Department of Community Medicine, UiT The Arctic University of Norway.

The participants could alternatively use a web-based questionnaire by logging onto a server administered by Norwegian Social Science Data Services (NSD), using a unique access code assigned to each participant. The content of the web questionnaire corresponded to the paper version, though the layout was different due to limitations in the web design system.

Together with relevant and required information about the survey, the questionnaires were sent out from Statistics Norway during the period from 9–12 January 2012. Two reminders were sent to non-responders after 6 weeks and after 4 months. The reminders contained information on the web-based questionnaire with its web address and each participant's unique access code, as well as contact information in case they wanted a new questionnaire. Although 50% of the participants sent in their questionnaire within the first 2 weeks, questionnaires continued to arrive for about 10 months. The deadline was set at 25 October, after which the data file was prepared for use. Five questionnaires that arrived after this date were excluded.

Information about the study was also provided to the public via the mass media and the project web page. In addition, posters were distributed to all local councils with information about the study encouraging people to participate.

The Norwegian questionnaires returned by post were manually clarified before digital scanning, whereas questionnaires filled out in a Sami language were entered by hand. Data from the web-based questionnaire were merged with the data from the questionnaires returned by post. At the end of the data collection, information on year of birth, place of residence and sex were issued from the Norwegian National Population Register and linked by Statistics Norway to the data file. The complete data file is stored and administered de-identified in EUTRO, a data storage system developed and administered by the Department of Community Medicine, UiT The Arctic University of Norway (16).

### Statistics

SAS statistical software for Windows version 9.2 (SAS Institute Inc., Cary, NC, USA) was used for both data management and statistical analysis. The data presented in this paper are primarily of a descriptive character, with some statistical tests between categorical variables using chi-square testing.

### Ethics

The data collection and storage was approved by the Norwegian Data Protection Authority (Datatilsynet), while all further use of the SAMINOR 2 data will require approval from the Regional Committee for Medical and Health Research Ethics for Northern Norway (REK-Nord) for each sub-project.

## Results

Among the 44,669 invitations, 1,424 letters were returned to sender, due to the wrong address or the recipient having moved. Since the people concerned never received the invitation letter, they were not considered to have been invited to participate. Altogether, 11,600 gave informed consent and participated in this study, which constitutes 27% of those invited. Table I shows an increased participation by age, with women participating to a greater extent than men. This gender effect was largest in the youngest age group, with a factor of 2 between the sexes.

**Table I:** Participation by age and gender: the SAMINOR 2 questionnaire study (n=11,600)

	Age (years)	Sample	Participants	%
Males	18–19	966	100	10.4
	20–29	3,987	426	10.7
	30–39	3,778	680	18.0
	40–49	4,876	1,096	22.5
	50–59	4,592	1,339	29.2
	60–69	4,366	1,508	34.5
	Total men	22,565	5,149	22.8
Females	18–19	844	173	20.5
	20–29	3,610	785	21.8
	30–39	3,586	965	26.9
	40–49	4,586	1,548	33.8
	50–59	4,236	1,594	37.6
	60–69	3,818	1,386	36.3
	Total women	20,680	6,451	31.2
All		43,245	11,600	26.8

A total of 1,842 participants (15.9%) chose the web-based questionnaire, 272 (2.3%) used the questionnaire in the Northern Sami language, 6 in Lule Sami and 6 in Southern Sami, while the remaining 9,474 participants chose the Norwegian paper questionnaire. The use of the web-based questionnaire increased after the reminders were sent out. More young people chose the web-based questionnaire, amounting to 1 in 4 below the age of 40, while only 1 of 8 in the 40–69 age group did the same. The usage of Sami languages was low (2.4%) and was approximately the same across the various age groups (data not shown). There was a small gender difference, with more men than women choosing the web-based questionnaire, while more women than men used Sami language questionnaires.

**Fig. 2:**
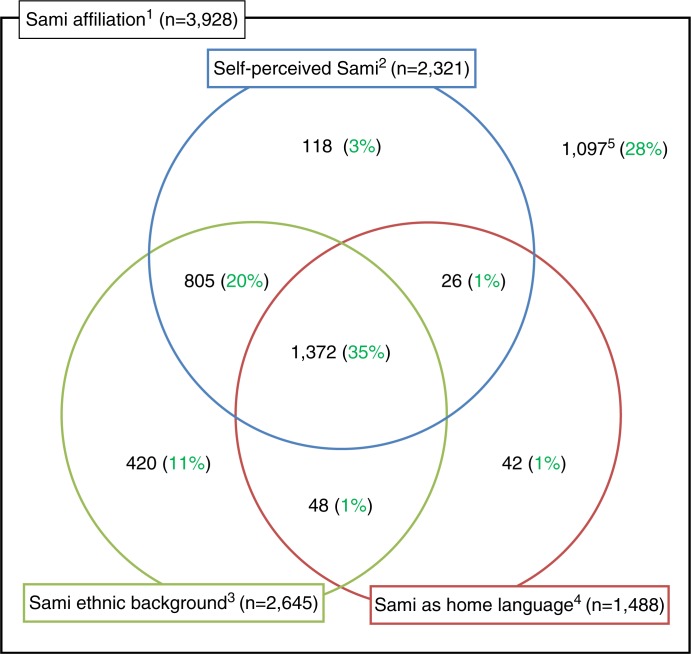
Distribution of sub-populations among participants with Sami affiliation: The SAMINOR 2 questionnaire study. ^1^ *Sami affiliation* is defined as Sami language being spoken at home by at least one of the grandparents, parents or the respondent, or Sami ethnic background reported for respondent or a parent, or that the respondent considers himself/herself as Sami. ^2^ *Self-perceived Sami* is defined as yes to the question: I *consider myself Sami*. ^3^ *Sami ethnic background* is defined as yes to the question: *My ethnic background is Sami*. ^4^ *Sami as home language* is defined as yes to the question: *My home language is Sami*. ^5^Respondents who reported use of the Sami language or ethnicity for grandparents or parents, but did not consider themselves to be Sami or have a Sami background/home language.

In total, 3,928 (34.1%) of the participants had some type of Sami affiliation, and of these, 59.1% reported that they considered themselves to be Sami (Fig. 2).

Participation by municipality units is shown in Table II, and overall, some geographical variations in participation were observed, ranging from just below 20% to slightly more than 35%.

**Table II:** Participation by municipality: the SAMINOR 2 questionnaire study

County	Municipality	Sample	Participants	%
Finnmark	Sør-Varanger	6,300	1,731	27.5
	Nesseby	568	151	26.6
	Tana	1,885	544	28.9
	Lebesby	856	224	26.2
	Karasjok	1,796	505	28.1
	Porsanger	2,663	690	25.9
	Kvalsund	625	169	27.0
	Loppa	674	186	27.6
	Alta	12,153	3,236	26.6
	Kautokeino	1,875	527	28.1
Troms	Kvænagen	810	204	25.2
	Kåfjord	1,409	361	25.6
	Storfjord	1,240	388	31.3
	Lyngen	1,902	534	28.1
	Lavangen	609	152	24.9
	Skånland	1,937	450	23.2
Nordland	Evenes	862	250	29.0
	Narvik	1,053	209	19.9
	Tysfjord	1,252	245	19.6
	Hattfjelldal	656	193	29.4
	Grane	52	12	23.1
Nord-Trøndelag	Namskogen	532	133	25.0
	Røyrvik	313	98	31.3
	Snåsa	820	288	35.1
Sør-Trøndelag	Røros	403	116	28.8
	Total	43,245	11,600	26.8

Table III presents characteristics of early and late responders, which are defined by response before or after the date when the first reminder was sent out. The late responders were associated with a younger age, Sami ethnicity, lower gross income and education. No gender differences were found.

**Table III:** Selected variables by early and late response: the SAMINOR 2 questionnaire study

		Early response		Late response		
				
		n	%	n	%	*p* a
Age (in years)	18–19	180	2.1	93	3.1	<0.0001
	20–29	783	9.2	428	14.1	
	30–39	1,130	13.2	515	16.9	
	40–49	1,996	23.3	648	21.3	
	50–59	2,253	26.3	680	22.4	
	60–69	2,215	25.9	679	22.3	
	Total	8,557		3,043		
Gender	Males	3,772	44.1	1,377	45.3	0.26
	Females	4,785	55.9	1,666	54.8	
Gross income^b^	<150,000	248	3.0	171	5.9	<0.0001
	150–300,000	842	10.2	372	12.8	
	301–450,000	1,482	18.0	580	20.0	
	451–600,000	1,610	19.5	573	19.7	
	601–750,000	1,298	15.7	408	14.0	
	751–900,000	1,535	18.6	426	14.7	
	>900,000	1,237	15.0	375	12.9	
	Total	8,252		2,905		
Education (in years)	<7	197	2.3	104	3.5	0.0011
	7–9	1,050	12.5	406	13.7	
	10–12	2,272	27.0	818	27.6	
	13–16	2,965	35.2	976	33.0	
	>16	1,942	23.1	657	22.2	
	Total	8,426		2,961		
Ethnic distribution	Sami	2,701	31.8	1,227	40.8	<0.0001
	Non-Sami	5,795	68.2	1,782	59.2	
	Total	8,496		3,009		

a Pearson's chi-square test for difference between early and late responders.

b In Norwegian krone, NOK.

To assess any potential selection bias, we studied the sub-sample of people who participated in the SAMINOR 1 study and who were also invited to the SAMINOR 2 questionnaire study. SAMINOR 2 participants were compared to non-participants based on their answers to some key characteristics in SAMINOR 1. Table IV shows that a selection bias by age, gender, income and education was likely. Although participation seemed to be unaffected by ethnicity, when dividing the group with an ethnic Sami background into those with and without self-perceived Sami ethnicity, we found that those who considered themselves to be Sami participated to a slightly greater extent (data not shown).

**Table IV:** Selected variables from the first SAMINOR study (2003–04) by participation in the SAMINOR 2 questionnaire study (2012)^a^

		Non-participants		Participants		
				
		n	%	n	%	*p* b
Age (years) in 2012	43–49	1,320	21.1	872	19.1	0.035
	50–59	2,414	38.5	1,772	38.9	
	60–69	2,530	40.4	1,916	42.0	
	Total	6,264		4,560		
Gender	Males	3,141	50.1	2,067	45.3	<0.0001
	Females	3,123	49.9	2,493	54.7	
Gross income^c^	<150,000	403	7.1	166	3.8	<0.0001
	150–300,000	1,367	24.1	834	19.2	
	301–450,000	1,810	31.8	1,275	29.4	
	451–600,000	1,421	25.0	1,295	29.8	
	601–750,000	470	8.3	539	12.4	
	>750,000	212	3.7	231	5.3	
	Total	5,683		4,340		
Education (in years)	<7	119	2.1	63	1.5	<0.0001
	7–9	1,599	28.1	772	18.2	
	10–12	2,111	37.1	1,372	32.3	
	13–16	1,288	22.7	1,251	29.4	
	>16	568	10.0	790	18.6	
	Total	5,685		4,248		
Ethnic distribution	Sami	2,187	35.7	1,681	37.1	0.20
	Non-Sami	3,934	64.3	2,852	62.9	
	Total	6,121		4,533		

a Sample restricted to 10,824 subjects aged 43–69, who were invited to the SAMINOR 2 questionnaire study and also participated in SAMINOR 1.

b Pearson's chi-square test for differences between participants and non-participants.

c In Norwegian kroner, NOK.

## Discussion

We have described the data collection and participation in the SAMINOR 2 questionnaire study. The main observation was a low response rate, especially among the younger population. This is in accordance with recently observed trends in population-based surveys in Norway (17, 18), as well as internationally, that is, people's willingness to participate in questionnaire-based studies has declined in recent years (19). Our response rate, both with respect to magnitude and pattern with decreased participation among men and the younger part of the invited sample, is in agreement with other comparable epidemiological surveys (18).

The response rate achieved raises the question of external validity. However, due to strict regulations for access to register-based data for non-responder analyses, limited knowledge about the non-responders curtails the ability to elucidate on this, with exceptions for the variables of age, sex and municipality. Information drawn from the SAMINOR 1 questionnaire is also of some value when assessing external validity despite the fact that only 25% of the invited sample in SAMINOR 2 participated in SAMINOR 1. The observation of no apparent selection bias by ethnic group must be interpreted with caution because the lack of ethnic registry data from the total source population hampers the ability to assess whether the distribution of ethnicity in the SAMINOR study reflects that of the actual population in this geographical area.

In future analyses and work with this SAMINOR 2 data set, potential selection bias and the generalizability issue need to be carefully addressed and discussed in relation to each specific theme under study.

As an alternative to the traditional questionnaire form, the option of a web-based questionnaire was not chosen by the participants to the extent we had expected. The observation that only approximately 16% of the total submitted questionnaires were digital appeared quite surprising. Even so, web-based questionnaires have only been utilized to a limited degree in population-based surveys in Norway, and to the best of our knowledge have not been used in larger epidemiological surveys of the general population. As expected, a larger proportion of the younger participants used the web-based solution. Nevertheless, we had expected that the web solution would be preferred to the paper form by the majority of the participants. Uncertainty regarding data safety could be a potential explanation for the refusal by the vast majority of the participants to report, for example, sensitive personal health information using the web questionnaire.

Data from Statistics Norway has revealed that 90% of the households in northern Norway have access to the Internet (20). In a user survey by the Norwegian Broadcasting Corporation's Sami Radio in 2009, 92% of the sample answered that they had used the Internet during the past 30 days (21). Unquestionably, web-based questionnaire tools have potential methodological advantages, namely that they save resources and that the accuracy of the data is increased. In addition, more research is needed on peoples’ perceptions of using web-based questionnaires in order to develop useful tools for data collection in population-based research.

The survey was marketed via the media, advertisements and information material. However, our observation of a very low participation rate among younger people indicates a challenge in marketing campaigns for such a broad age span (18–69 years). It would seem that our ambition of encouraging participation did not appeal to the younger group in particular. For future investigations aiming at reaching the younger adult generation, targeted designs in both the research tools and the information strategies are recommended (22).

The options for reporting ethnicity in the questionnaire were equal to those used in the SAMINOR 1 study in 2003/4. Due to its diverse nature, both the classification of ethnicity and its use as an independent variable in epidemiological research is complex and somewhat controversial (23). We recommend that in further analysis of the data from the SAMINOR study the inclusion criteria for Sami ethnicity are transparently explained and a priori classified for each research theme/question under study.

Despite the limitations of the SAMINOR 2 study, in particular in relation to low response rates, the SAMINOR 2 questionnaire study provides a unique database for research on a broad spectrum of highly relevant aspects of health and living conditions. It can also constitute the basis for further follow-up studies, for example, on selected research themes. The data was sampled from the general population in a large geographical area in Northern and Mid Norway. In addition, the reported ethnicity on an individual level, including various types of ethnic affiliation, enables a more comprehensive analysis across ethnic groups. Hence, in-depth knowledge of health and living conditions in the Sami population in Norway can be obtained. In addition, the linkage with the SAMINOR 1 study provides a further unique opportunity for studying trends in both diseases and risk factors in the rural areas of Central and Northern Norway. Finally, there will be even more unique possibilities to link this questionnaire-based study with the clinical study, which will be finalized June 2014.

In conclusion, knowledge generated from future theme-specific research utilizing the SAMINOR 2 database has the potential to benefit the general population in this geographical area of Norway, and the Sami people in particular, by providing scientific-based insight into health and living conditions in the multi-ethnic population in these parts of Norway.
